# Purification and characterization of hydroquinone dioxygenase from *Sphingomonas *sp. strain TTNP3

**DOI:** 10.1186/2191-0855-1-8

**Published:** 2011-05-27

**Authors:** Boris A Kolvenbach, Markus Lenz, Dirk Benndorf, Erdmann Rapp, Jan Fousek, Cestmir Vlcek, Andreas Schäffer, Frédéric LP Gabriel, Hans-Peter E Kohler, Philippe FX Corvini

**Affiliations:** 1 Institute for Ecopreneurship, School of Life Sciences, University of Applied Sciences Northwestern Switzerland, Muttenz, Switzerland; 2 Institute for Environmental Research, Rheinisch-Westfälische Technische Hochschule, Aachen, Germany; 3 Bioprocess Engineering, Otto von Guericke University, Magdeburg, Germany; 4 Bioprocess Engineering, Max Planck Institute for Dynamics of Complex Technical Systems, Magdeburg, Germany; 5 Institute of Molecular Genetics, Academy of Sciences of the Czech Republic, Prague, Czech Republic; 6 Centre for Applied Genomics, Prague, Czech Republic; 7 Institute of Clinical Chemistry and Laboratory Medicine, University of Rostock, Rostock, Germany; 8 Swiss Federal Institute of Aquatic Science and Technology, Dübendorf, Switzerland; 9 School of the Environment, Nanjing University, Nanjing, China

**Keywords:** hydroquinone, dioxygenase, Sphingomonas, nonylphenol, bisphenol A

## Abstract

Hydroquinone-1,2-dioxygenase, an enzyme involved in the degradation of alkylphenols in *Sphingomonas *sp. strain TTNP3 was purified to apparent homogeneity. The extradiol dioxygenase catalyzed the ring fission of hydroquinone to 4-hydroxymuconic semialdehyde and the degradation of chlorinated and several alkylated hydroquinones. The activity of 1 mg of the purified enzyme with unsubstituted hydroquinone was 6.1 μmol per minute, the apparent K_m _2.2 μM. ICP-MS analysis revealed an iron content of 1.4 moles per mole enzyme. The enzyme lost activity upon exposure to oxygen, but could be reactivated by Fe(II) in presence of ascorbate. SDS-PAGE analysis of the purified enzyme yielded two bands of an apparent size of 38 kDa and 19 kDa, respectively. Data from MALDI-TOF analyses of peptides of the respective bands matched with the deduced amino acid sequences of two neighboring open reading frames found in genomic DNA of *Sphingomonas *sp strain TTNP3. The deduced amino acid sequences showed 62% and 47% identity to the large and small subunit of hydroquinone dioxygenase from *Pseudomonas fluorescens *strain ACB, respectively. This heterotetrameric enzyme is the first of its kind found in a strain of the genus *Sphingomonas sensu latu*.

## Introduction

Both *Sphingomonas *sp. strain TTNP3 and *Sphingobium xenophagum *Bayram are able to degrade several branched isomers of nonylphenol and bisphenol A, well-known endocrine disruptors, by *ipso *substitution. i.e. *ipso*-hydroxylation and subsequent detachment of the side chain of the alkylphenol. In these pathways hydroquinone is formed as a key metabolite (Kolvenbach et al. 2007; Corvini et al. 2006; Gabriel et al. 2007a; Gabriel et al. 2007b; Gabriel et al. 2005). Hydroquinone (HQ) is also a key intermediate in the degradation of several other compounds of environmental importance, such as 4-nitrophenol (Spain and Gibson 1991), γ-hexachlorocyclohexane (Miyauchi et al. 1999), 4-hydroxyacetophenone (Moonen et al. 2008a) and 4-aminophenol (Takenaka et al. 2003).

There are two established pathways in the literature for the degradation of hydroquinone. One involves direct ring cleavage of hydroquinone by dioxygenases containing Fe(II) in their active center, resulting in the formation of 4-hydroxymuconic acid semialdehyde (HMSA) (Chauhan et al. 2000; Miyauchi et al. 1999; Moonen et al. 2008b). The second pathway requires the hydroxylation of hydroquinone to benzene-1,2,4-triol (Eppink et al. 2000) which is then cleaved to yield maleylacetic acid (Rieble et al. 1994; Jain et al. 1994) by dioxygenases containing Fe(III) in their active center (Latus et al. 1995; Travkin et al. 1997; Ferraroni et al. 2005).

The hydroquinone dioxygenases (HQDO) can be divided into two subtypes that have few similarities. Members of type I are phylogenetically related to the well-described extradiol catechol dioxygenases, (Eltis and Bolin 1996) and are monomeric (Xu et al. 1999). Moreover, they are involved in the degradation of HQ and chlorinated HQ formed during degradation of pentachlorophenol and γ-hexachlorocyclohexane by several members of the *Sphingomonas *genus (Cai and Xun 2002; Miyauchi et al. 1999; Lal et al. 2010). Supposedly, more homologs exist as DNA sequences with similarities of 99% and higher to the PcpA encoding sequence have been attributed to γ-hexachlorocyclohexane degradation in other sphingomonads, i.e. strains of the genus *Sphingomonas sensu latu *(Dogra et al. 2004; Manickam et al. 2008; Yamamoto et al. 2009; Lal et al. 2010) and nitrophenol degradation in *Cupriavidus necator *Jmp134(Yin and Zhou 2010). Type II dioxygenases consist of two different subunits forming an α2β2 heterotetramer. These enzymes are responsible for ring cleavage of HQ formed during degradation in the degradation pathway of hydroxyacetophenone (Moonen et al. 2008b) and in the degradation pathway of *p*-nitrophenol (Wei et al. 2010; Zhang et al. 2009; Shen et al. 2010). Interestingly, members of type II have not been found in sphingomonad strains yet.

Recently, PcpA, a type I HQDO from *Sphingobium chlorophenolicum*, has been subjected to homology based structural modeling in combination with site directed mutagenesis, yielding information on the native tertiary structure and the histidine residues responsible for chelating the Fe(II) in the active center (Machonkin et al. 2009). However little is known about HQDO in general, as until now only the HQDO from *Pseudomonas fluorescens *strain ACB has been purified and thoroughly characterized (Moonen et al. 2008b).

Here, we describe the purification and the properties of a novel type II heterotetrameric HQDO that we isolated from *Sphingomonas *sp. strain TTNP3.

## Materials and methods

### Materials

Tris, ammonium sulfate, ascorbic acid were purchased from Applichem (Axon Lab, Baden-Dättwil, Switzerland), hydroquinone and technical grade nonylphenol were purchased from Fluka (Buchs, Switzerland). Standard I Medium was purchased from Merck (Zug, Switzerland). Methylhydroquinone was obtained from Sigma (Buchs, Switzerland), ethylhydroquinone and t-butylhydroquinone were obtained from ACBR (Karlsruhe, Germany), propyl-, pentyl- and hexylhydroquinone were obtained from Labotest (Niederschöna, Germany). 2-(1-methyl-1-octyl)-hydroquinone was synthesized by Friedel-Crafts alkylation from hydroquinone with 2-nonanol obtained from Sigma (Buchs, Switzerland) according to the protocol of Corvini *et al: *(Corvini et al. 2004b). All other chemicals were of analytical grade. All columns used for protein purification were purchased from GE Healthcare (Uppsala, Sweden).

### Bacterial strains and culture conditions

*Sphingomonas *sp. strain TTNP3 was obtained from Professor Willy Verstraete (LabMet, University Ghent, Belgium). The strain was grown on Standard I Medium as described previously (Corvini et al. 2004c). Enzymatic activity was induced by the addition of 0.5 mM technical grade nonylphenol 16 hours prior to harvesting the cells at an OD_550 _of about 3.0. Cultures were then centrifuged at 4,500 * *g *for 15 minutes, resuspended in 50 mM Tris, pH 7.5 at 4°C. This washing procedure was repeated twice. In the last step, the cells were resuspended to an OD_550 _of 60 and stored at -20°C.

### Sequence data

DNA analysis of *Sphingomonas *sp. strain TTNP3 was performed with data obtained from genome shotgun sequencing.

### Nucleotide sequence accession number

The nucleotide and amino acid sequence data reported in this paper have been deposited in the GenBank sequence database under accession number JF440299.

### Purification of HQDO from strain TTNP3

Purification steps were performed on a Pharmacia FPLC liquid chromatography system. All steps were performed at 4°C, unless stated otherwise. Buffers for purification were stored under argon (Messer AG, Switzerland). Thawed cells were diluted to an OD_550 _of 20 in 16 mL 50 mM Tris, pH 7.5, 4-hydroxybenzoic acid (HBA, 1 M in Ethanol) and ascorbic acid (0.5 M dissolved in equimolar NaOH) were added to final concentration of 0.5 mM and 2.5 mM, respectively. Cells were disrupted by sonication on ice (20 minutes at 100% intensity, 0.6 s/s duty cycle using a Labsonic M sonicator by B. Braun Biotech, equipped with a 3 mm probe). After centrifugation (21,500 * *g *for 15 min), five preparations of cell extract were pooled to a volume of 65 mL and subjected to ammonium sulfate precipitation, by adding ammonium sulfate to 40% saturation with subsequent centrifugation at 21,500 * *g *for 30 min. The supernatant was diluted to 20% ammonium sulfate saturation with 50 mM Tris, pH 7.5, containing 0.5 mM HBA (buffer A) and loaded onto two coupled Phenyl Sepharose High Performance columns with a total volume of 10 mL, previously equilibrated with buffer A containing 20% ammonium sulfate (buffer B). After washing with 40 mL of buffer B, HQDO activity was eluted by applying a linear gradient from 100% buffer B to 100% buffer A in 100 mL. Active fractions were pooled and desalted over 4 coupled Hi Trap Desalting columns (total volume of 20 mL), equilibrated with buffer A, and then applied to a 20 mL DEAE column. After washing with 40 mL buffer A, proteins were eluted with a linear gradient from 0 to 400 mM NaCl in 200 mL buffer A. Active fractions were desalted as described above and loaded onto a Mono Q column. After washing with 10 mL buffer A, activity was eluted with a linear gradient from 0 to 1 M NaCl in 40 mL buffer A and stored at -20°C under argon. Size exclusion chromatography of the native enzyme was carried out on a HP Agilent Series 1050 HPLC system (Agilent Technologies, Basel, Switzerland) equipped with a Superose 6 column equilibrated with phosphate buffer (10 mM, pH 7.0) containing 137 mM NaCl. The system was calibrated with a standard mixture of thyroglobulin, myosin, ovalbumin, RNAse A and aprotinin (Sigma, Switzerland) and detection was carried out at 280 nm

### Enzyme activity

Enzyme activity was routinely measured at 25°C by measuring the formation of HMSA at 320 nm (ε_320 _= 11000 M^-1 ^* cm^-1 ^(Spain and Gibson 1991)) on a Synergy 2 multi-mode microplate reader (Biotek, Luzern, Switzerland). The assay mixture (250 μL) typically contained ca. 50 nM enzyme solution in 250 μL air saturated 50 mM Tris, pH 7.0, reactions were started by the addition of 100 μL freshly prepared solution of 350 μM HQ in 50 mM Tris buffer, pH 7.0, resulting in a final substrate concentration of 100 μM. Activity of HQDO on substituted hydroquinones was determined by measuring oxygen consumption with a Clarke type oxygen electrode (Oxytherm system, Hansatech, Reutlingen, Germany). To a total volume of 800 μL, about 100 nM of enzyme was added before the addition of 8 μL of an ethanolic solution of 20 mM substrate to reach a final substrate concentration of 200 μM.

As the enzyme was subject to suicide deactivation upon incubation with HQ, only initial rates recorded within 20 seconds after the addition of substrate were used for determination of kinetics. k_M _was determined by Prism version 5.02(GraphPad).

### Enzyme stability

The stability of HQDO at 30°C was studied by incubating the purified enzyme in 50 mM Tris buffer, pH 7.0 at 30°C in absence of 4-HBA under argon and, in the presence and absence of 0.5 mM 4-HBA under normal atmosphere, respectively.

### Enzyme inactivation by iron chelators

The inactivation of HQDO was determined by incubation of the purified enzyme at 30°C in the presence of 0.1 mM and 1 mM 2,2'-bipyridyl, 0.1 mM and 1 mM *o*-phenanthroline, respectively, before testing for remaining activity after 15 minutes. The purified enzyme was also incubated at 30°C in the presence of 0.1 mM hydrogen peroxide, before assaying for remaining activity after one minute.

### Protein content/SDS-PAGE

Protein content was determined using the Bio-Rad Protein Assay (Biorad) using lysozyme as a standard. Sodium dodecyl sulfate-polyacrylamide gel electrophoresis (SDS-PAGE) was carried out with 15% Tris-glycine minigels according to a standard protocol (Laemmli 1970) in a Mini-PROTEAN Tetra Cell (BioRad).

### ICP-MS

Iron concentrations in fractions eluting from the MonoQ columns were determined using an inductively coupled plasma-mass spectrometry (ICP-MS) system (Agilent 7500cx) equipped with an Octopole Reaction System. Water and hydrochloric acid were added to 750 μL of each fraction to a total volume of 2 mL and a HCl concentration of 1.5%, before measuring the samples on the inductively coupled plasma-mass spectrometry system. The measurements were performed using a radio frequency power of 1500W, a carrier gas flow of 0.79 L/min, a make-up gas flow of 0.30 L/min at a sample depth of 8 mm. Fe was quantified on *m/z *= 56 whereas *m/z *= 57 served as control to verify quantification results. Other elements assayed were Mg (*m/z *= 24), Mn (*m/z *= 55), Ni (*m/z *= 60). All measurements were carried out in collision mode with an optimized helium flow of 5 mL/min. Indium served as internal standard.

### GC-MS

Samples for GC-MS analysis were acidified with a drop of 6 M HCl and extracted with two volumes of ethyl acetate three times; the organic phase was dried over Na_2_SO_4 _before evaporation under a gentle nitrogen stream. Extracts were redissolved in acetonitrile/BSTFA (90:10 v/v) for derivatization at 75°C for 15 minutes. Samples were analyzed in an Agilent 7890A series gas chromatograph (Agilent Technologies, Basel, Switzerland) equipped with a Zebron ZB-5MS column, (30 m by 0.25 mm, 0.25 μm film thickness, Phenomenex) coupled to an Agilent 5975C series mass spectrometer. The mass selective detector (EI) was operated in the scan mode (mass range *m/z *50-600) with an electron energy of 70 eV. The temperature program was 70°C for 3 min, 8°C per minute to 250°C; the injector temperature was 90°C; the interface temperature 280°C. The injection volume was 1 μL (split 1:30). The carrier gas was helium (1 mL/min).

### Protein identification

Briefly, protein bands were picked from the SDS gel. The proteins were digested tryptically in gel and identified by nanoHPLC-nanoESI-MS/MS. Fully automated online pre-concentration and separation of the tryptically digested samples was performed using a set of capillary- and nanoHPLC instruments of the 1100 Series (Agilent, Waldbronn, Germany) operated in series. Mass spectrometric detection was carried out by online coupling nanoHPLC with a QSTAR XL (QqTOF) mass spectrometer (Applied Biosystems/MDS/Sciex, Darmstadt, Germany) operated in MS and MS/MS mode. The instrument was equipped with an online nano-electrospray ion source (NanoSpray II Source) and upgraded with a heated interface (Vester et al. 2009).

A first data interpretation of acquired product-ion spectra of the nanoHPLC-nanoESI-MS/MS analysis, was performed by an automatic database search with MASCOT™ (version 2.2, Matrix Science, London, UK) (Perkins et al. 1999). For all searches, the MASCOT peptide fragmentation mass fingerprint algorithm screening against all species of the actual NCBI non-redundant database (2010-04-20) was used to identify the corresponding peptides. A detailed description of this procedure was previously reported (Vester et al. 2009). Additionally, most abundant peptides were selected and manually *de novo *sequenced using an in-house software tool.

### Phylogenetic analysis of HqdA and HqdB

A phylogenetic tree of HqdA and HqbB found in *Sphingomonas *sp. strain TTNP3 and respectively corresponding sequences from 21 other bacterial strains that were found to be similar by BLAST analysis was constructed by rendering a ClustalX 2 alignment and using Treeview 1.6.6

## Results

### Purification of HQDO from *Sphingomonas *sp. strain TTNP3

**Figure 1:**
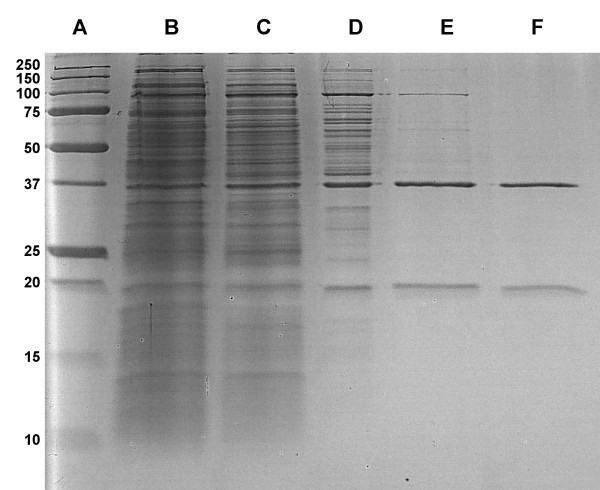
**SDS-PAGE of HQDO from *Sphingomonas *sp. strain TTNP3**. Lane A, marker proteins: lane B, crude cell extract; lane C, ammonium sulfate fractionation supernatant; lane D, phenyl-Sepharose pool; lane E, DEAE pool; lane F; MonoQ pool.

Even though strain TTNP3 appears to express the HQ cleaving enzyme constitutively (Corvini et al. 2006), higher amounts of enzyme activity could be achieved by inducing the cells with technical nonylphenol mixture prior to harvesting them. Without the addition of a reversible inhibitor, HQDO lost activity rapidly, impeding success of early purification attempts. Table 1 presents the result of a typical preparation of purified enzyme from 8 g of cells. Purification in four steps typically resulted in a yield of 30%, a purification factor of 42 and a specific activity of 6.1 U mg^-1^. SDS-PAGE analysis showed the presence of two major protein bands, corresponding to masses of 38 kDa and 19 kDa, respectively (Figure 1). The purified enzyme eluted from the Superose 6 column in one symmetrical peak with an apparent molecular mass of 120 kDa (data not shown).

**Table 1:** Purification scheme for HQDO from *Sphingomonas *sp. strain TTNP3

Purification step	Activity (U)	Protein (mg)	Spec. act. (U mg^-1^)	Purification factor	Yield (%)
Cell extract	35.7	245	0.15	1	100
Ammonium sulfate fractionation	35.9	108	0.33	2.3	101
Phenyl-Sepharose	34.5	19.2	1.80	12.4	89
DEAE	14.8	3.3	4.42	30.4	44
MonoQ	9.5	1.6	6.06	41.6	30

### Physico-chemical properties of the enzyme

ICP-MS analysis of the fractions eluting from the final purification step, i.e. MonoQ column, revealed a clear correlation between the enzyme activity in the fraction and its respective iron content. Based on the apparent molecular mass of 120 kDa, 1 μmol of enzyme contained 1.4 μmol of iron and 0.04 μmol of manganese. Other metal species could not be attributed to fractions containing enzyme activity. HQDO showed an absorption maximum at 279 nm, slight absorption between 300 nm and 400 nm, yet none longer wavelengths.

### Catalytic properties

HQDO from *Sphingomonas *sp. strain TTNP3 catalyzed the ring cleavage of hydroquinone to HMSA under consumption of an equimolar amount of molecular oxygen (data not shown). Maximal enzyme activity was observed between pH 7 and pH 8. The apparent K_m _for HQ was determined to be 2.2 μM with a standard error of. 0.2. k_cat _was determined to be 811 min^-1 ^with a standard error of 15 for the heterotetrameric enzyme and kÂ¬_cat_/k_M _was determined to be 369 min^-1^.

HQDO was shown to readily lose activity upon incubation with its substrate, HQ. Inactivation of the enzyme appeared to be irreversible, as enzyme activity could not be restored by incubation with Fe(II) ions (compare Enzyme stability). Nevertheless, fresh enzyme added to a spent reaction mixture transformed the substrate at the normal rate.

Besides acting on hydroquinone as a substrate, HQDO catalyzed the conversion of several other substituted hydroquinones (Table 2). Phenol, catechol, resorcinol and 4-mercaptophenol were not used as substrate by the enzyme (data not shown).

**Table 2:** Substrate specificity of HQDO of *Sphingomonas *sp. strain TTNP3 (relative rate of oxygen consumption with 200 μM substrate compared to HQ as substrate)

Substrate (200 μM)	Activity (%)	SD (%)
Hydroquinone	100	12.8
Chlorohydroquinone	29	0.8
2-Methoxyhydroquinone	59	6.7
2-Methylhydroquinone	139	9.3
2-Ethylhydroquinone	83	4.3
2-Propylhydroquinone	23	2.6
2-t-Butylhydroquinone	5	0.6
2-Pentylhydroquinone	19	1.1
2-Hexylhydroquinone	<2	1.1
2-(1-methyl-1-octyl)-hydroquinone	<2	0.5

Enzyme activity was inhibited by the substrate analog 4-HBA. Inhibition was shown to be reversible, as samples showed normal reaction rates after removal of 4-HBA by gel filtration (data not shown). A number of other phenolic compounds inhibited the degradation reaction as well. The strongest inhibitions were observed with 4-hydroxybenzonitrile, 4-mercaptophenol, benzoquinone and vanillin (Table 3).

**Table 3:** Enzyme activity on HQ in the presence of phenolic inhibitors of HQDO

Inhibitor	Activity (%)	Inhibitor concentration (μM)	SD (%)
4-Hydroxybenzoate	46	200	0.4
3,4-Dihydroxybenzoate	94	200	4.6
4-Hydroxybenzylcyanide	<1	200	0.3
	3	20	0.3
	16	2	0.2
Aminobenzoic acid	93	200	1.2
Vanillin	7	200	2.2
	18	100	7.4
Vanillyl alcohol	62	200	1.0
Vanillate	86	200	1.0
4-Coumaric acid	97	200	1.9
Caffeic acid	98	80	2.5
Phenol	98	200	1.7
Catechol	93	200	1.3
Resorcinol	99	200	0.5
4-Nitrophenol	27	200	1.2
4-Mercaptophenol	1	200	
	5	20	
Benzoquinone	3	200	0.9
	16	20	0.3

### Product identification

**Figure 2:**
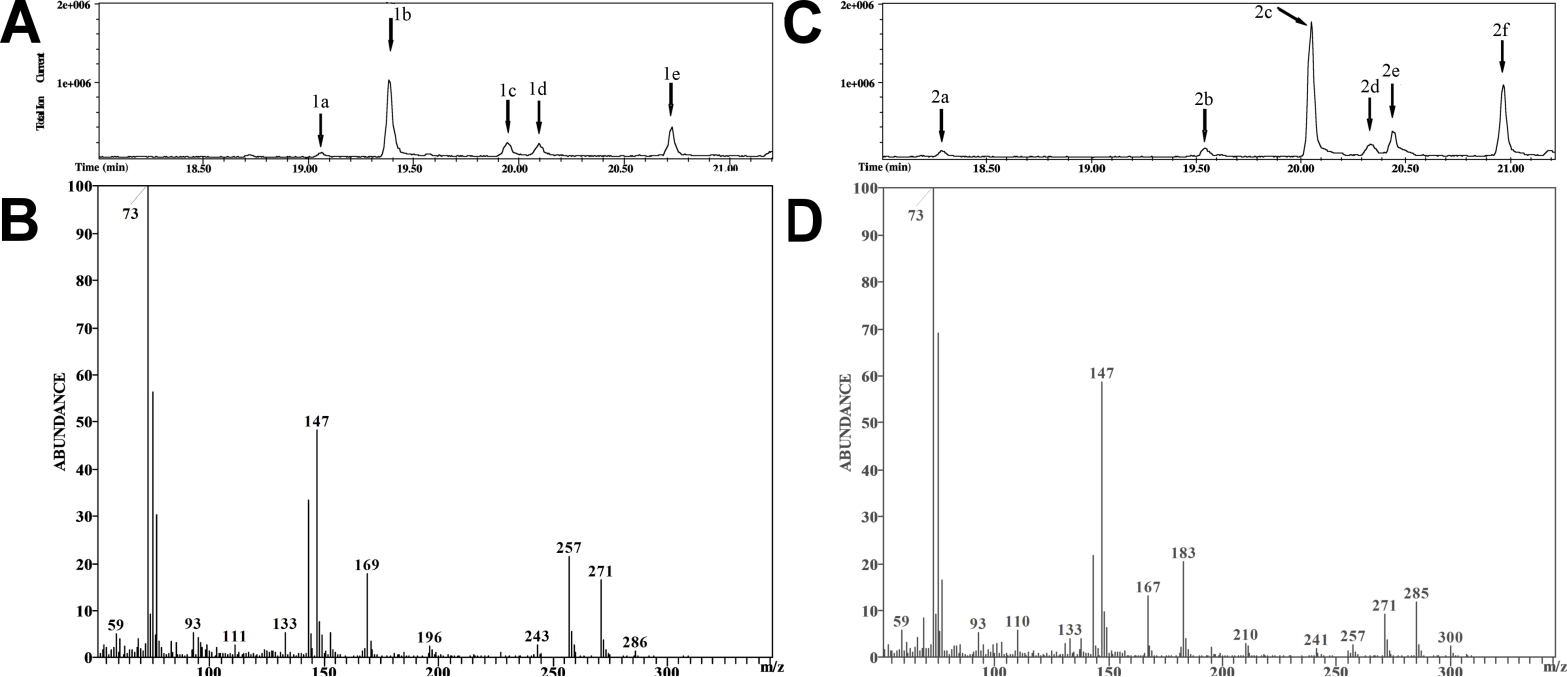
**A, GC-MS total ion chromatogram of the trimethylsilylated ring cleavage product of hydroquinone; B, mass spectrum of peak 1b from Figure 2A; C, GC-MS total ion chromatogram of the trimethylsilylated ring cleavage product of 2-methylhydroquinone; D, mass spectrum of the product peak 2c from Figure 2C**.

GC-MS analysis of the trimethylsilylated HQ ring cleavage products resulted in a chromatogram with five peaks that showed similar mass spectra (Figure 2A, peak 1b: *m/z *286 (M^+.^, 1.2%); 271 (M^+. ^- **^.^**CH_3_, 16.4%); 257 (M^+. ^- **^.^**CHO, 23.4%); 243 (2.4%); 196 (M^+. ^- **^.^**OSi(CH_3_)_3_, 2.1%); 169 (M^+. ^- **^. ^**Si(CH_3_)_3 _- CO_2_, 17.5%); 147 ([(Si(CH_3_)_3_)_2 _+ H]^+^, 48.1%); 143 (M^+. ^- **^. ^**Si(CH_3_)_3 _- CO_2 _- HC≡CH, 33, 33.3%); 93 (5.1%); 77 (30.1%); 75 (56.2%); 73 +Si(CH3)3, 100%, compare Table 4). Based on mass spectral analysis and published data (Miyauchi et al. 1999; Kohler et al. 1993), we identified the corresponding products as stereoisomers (*cis*-*trans*-isomers and conformers) of 4-hydroxmuconic acid semialdehyde (4-hydroxy-6-oxohexa-2,4-dienoic acid).

**Table 4:** Mass spectra of the detected peaks of trimethylsilylated HMSA (relative abundances in %)

peak	*t*_R _(min)	m/z														
		286	271	257	243	196	169	153	147	143	133	111	93	77	75	73
1a	19.07	4	10	33	n.d.	n.d.	21	5	11	16	2	4	15	100	72	85
1b	19.39	1	16	21	2	2	18	2	48	33	5	3	5	30	56	100
1c	19.95	1	29	10	6	6	66	16	29	13	3	3	7	45	59	100
1d	20.11	n.d.	15	13	5	9	99	12	27	9	7	5	10	32	47	100
1e	20.73	n.d.	33	5	4	4	39	14	21	28	4	3	7	26	43	100

Similarly, work-up and analysis of the cleavage products of methylhydroquinone showed a chromatogram with six peaks. The spectra corresponding to the two by far most intensive peaks were very similar, showing signals at *m/z *300 (M^+.^), 285 (M^+. ^- **^.^**CH_3_), 271 (M^+. ^- **^.^**CHO), 257 (M^+. ^- 43), 210, 183 (M^+. ^- **^. ^**Si(CH_3_)_3 _- CO_2_), 147 ([(Si(CH_3_)_3_)_2 _+ H]^+^), 143 (M^+. ^- **^. ^**Si(CH_3_)_3 _- CO_2 _- HC≡CCH_3_) (compare Figure 2C, peak 2c, Table 5 range above *m/z *140). Loss of a neutral mass of 29 amu is indicative of the presence of an aldehyde group. Combining this conclusion with a general mass spectral analysis and biochemical reasoning, we propose that the two major chromatographic peaks correspond to stereo or position isomers of trimethylsilylated methyl-4-hydroxymuconic acid semialdhyde (4-hydroxy-6-oxohexa-2,4-dienoic acid with a methyl substituent at positions 2, 3 or 5). Hence, ring cleavage proceeded between a C-OH and a neighboring C-H group of the methylhydroquinone substrate (and not between the neighboring C-OH and C-CH_3 _groups). Assuming that *m/z *143 ions were produced by loss of H-C≡C-CH_3 _(R_1_-C≡C-R_2_, see additional file 1) from *m/z *183 ions (M^+. ^- **^. ^**Si(CH_3_)_3 _- CO_2_) further restricts the possible cleavage sites to the ring bonds C(1) - C(6) and C(4)-C(5) in methylhydroquinone (cleavage of the C(3) -C(4) bond would have led to a loss of HC≡CH from the *m/z *183 ions).

**Table 5:** Mass spectra of the detected peaks of trimethylsilylated methyl-HMSA (relative abundances in %)

peak	*t*_R _(min)	m/z																	
		300	285	272	271	257	241	210	195	183	167	147	143	133	110	93	77	75	73
2a	18.29	n.d.	3	4	n.d.	6	n.d.	3	n.d.	59	12	40	5	6	6	11	30	78	100
2b	19.54	n.d.	7	n.d.	14	n.d.	2	6	3	26	11	81	4	8	11	12	34	100	87
2c	20.05	2	12	n.d.	9	2	2	3	2	20	13	58	22	4	6	5	16	69	100
2d	20.34	3	6	n.d.	5	5	1	10	5	33	20	15	8	3	4	5	17	41	100
2e	20.45	n.d.	4	6	n.d.	4	n.d.	n.d.	n.d.	11	3	59	3	3	3	3	12	100	75
2f	20.97	2	6	n.d.	1	2	n.d.	8	6	10	16	12	9	2	n.d.	4	8	35	100

### Enzyme stability

After incubation of the desalted enzyme at 30°C under normal atmosphere and without inhibitor for two hours, more than 20% of the initial activity was lost, while no loss of activity was observed when stored under argon or with the inhibitor 4-HBA, respectively. Incubation of 2.7 μM purified enzyme in presence of 0.5 mM inhibitor under argon atmosphere for 15 days, resulted in 16% and 9% loss of activity when kept at 0°C and 20°C, respectively. In the former case, incubation of the enzyme with 0.1 mM Fe_2_SO_4 _and 0.1 mM ascorbate on ice for 30 minutes prior to the assay could partially restore the activity, leaving a loss of 5% relative to the initial activity.

Part of the activity could be recovered by incubating the enzyme with 0.1 mM Fe_2_SO_4 _and 0.1 mM ascorbate on ice for 30 minutes prior to the assay.

*ortho*-Phenanthroline and 2,2'-dipyridyl, inactivated HQDO (Table 6). Rapid and complete inactivation also occurred upon incubation of the purified enzyme with the oxidizing agent hydrogen peroxide at 100 μM (Table 6).

**Table 6:** Inactivation of HQDO by iron(II) modifying substances

Inactivation substance	Substance concn (mM)	% activity after incubation at 30°C	SD (%)
*ortho*-phenanthroline	1	1%^a^	0.4
	0.1	22%^a^	1.0
2,2'-dipyridyl	1	23%^a^	2.9
	0.1	59%^a^	4.5
hydrogen peroxide	0.1	3%^b^	0.1

a Incubation time 15 min

b Incubation time 1 min

### Sequence data

**Figure 3:**
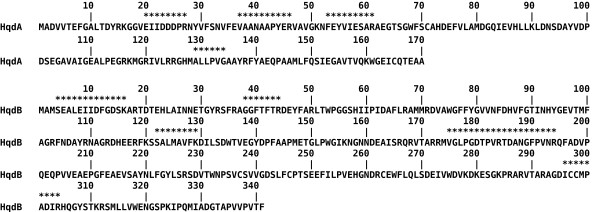
**Amino acid sequences deduced from open reading frames found in a part of genomic DNA from *Sphingomonas *sp. strain TTNP3**. The amino acids marked with asterisks were identified by nanoHPLC-nanoESI-MS/MS (from residues 175 to 194, two peptides were matched, one ranging from 175 to 184, the other one from 185 to 194).

nanoHPLC-nanoESI-MS/MS-analysis of bands resulting from SDS-PAGE of the purified enzyme and subsequent *de novo *sequencing yielded four peptides for the 19 kDa band, and six peptides for the 38 kDa band, respectively. These matched with amino acid sequences deduced from open reading frames that had been identified in genomic DNA from *Sphingomonas *sp. strain TTNP3, tentatively named HqdA and HqdB (Figure 3). A MASCOT search against a user database containing the sequences of HqdA and HqdB confirmed the identification for HqdA (Mowse Score: 435, sequence coverage: 30%) and HqdB (Mowse Score: 318, sequence coverage: 44%). HqdA and HqdB showed a sequence identity of 61% and 47% compared to the small and large subunit of HQDO from *Pseudomonas fluorescens *strain ACB, respectively.

**Figure 4:**
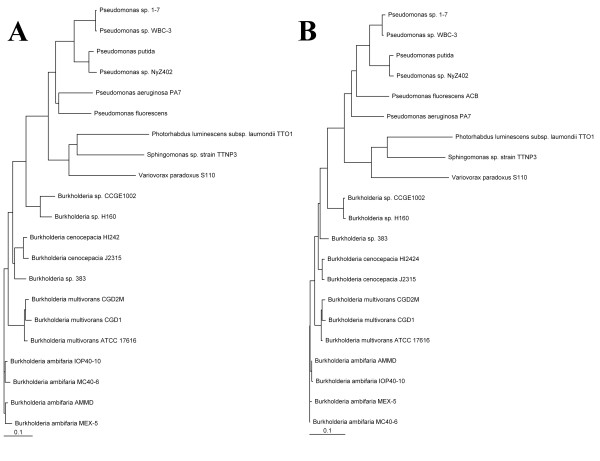
**Phylogenetic trees of the sequences of HqdA (A) and HqdB (B) and the respective homolog sequences from *Burkholderia *sp**. 383 (gi 78063587 and gi 78063586), *Burkholderia *sp. CCGE1002 (gi 295680998 and gi 295680997), *Burkholderia *sp. H160 (gi 209517843 and gi 209517844), *Burkholderia ambifaria *AMMD (gi 115359956 and gi 115359957), *Burkholderia ambifaria *IOP40-10 (gi 170700037 and gi 170700038), *Burkholderia ambifaria *MC40-6 (gi 172062406 and gi 172062407), *Burkholderia ambifaria *MEX-5 (gi 171319707 and gi 171319708), *Burkholderia cenocepacia *HI2424 (gi 116691528 and gi 116691529), *Burkholderia cenocepacia *J2315 (gi 206562327 and gi 206562328), *Burkholderia multivorans *ATCC 17616 (gi 161523095 and gi 161523094) *Burkholderia multivorans *CGD1 (gi 221212137 and gi 221212136), *Burkholderia multivorans *CGD2M (gi 221199017 and gi 221199016), *Burkholderia phymatum *STM815 (gi 186470422 and gi 186470423), *Photorhabdus luminescens *subsp. laumondii TTO1 (gi 37524165 and gi 37524166), *Pseudomonas aeruginosa *PA7 (gi 152988009 and gi 152987326), *Pseudomonas fluorescens *ACB (gi 182374631 and gi 182374632), *Pseudomonas putida *(gi 224460045 and gi 260103908), *Pseudomonas *sp. 1-7 (gi 284176971 and gi 284176972), *Pseudomonas *sp. NyZ402 (gi 269854714 and gi 269854713), *Pseudomonas *sp. WBC-3 (gi 156129389 and gi 156129388) and *Variovorax paradoxus *S110(gi 239820773 and gi 239820774). Sequences were retrieved from NCBI via BLAST search, subsequently aligned using ClustalX 2 and rendered using Treeview 1.6.6.

A dendrographic tree of HqdA and HqbC found in *S*. sp. strain TTNP3 and respectively corresponding sequences from 21 other bacterial strains that were found to be similar by BLAST analysis was constructed by amino acid sequence alignment via Clustal × version 2.0.11 (Larkin et al. 2007) and drawn by Treeview version 1.6.6 http://taxonomy.zoology.gla.ac.uk/rod/treeview.html (Figure 4). For complete multiple sequence alignments refer to the additional files 2 and 3.

## Discussion

We were able to isolate and characterize a protein from *Sphingomonas *sp. strain TTNP3 that catalyzes the Fe^2+^- and O_2_-dependent conversion of HQ to 4-hydroxymuconic semialdehyde. Like nonylphenol *ipso*-hydroxylases, i.e. the first enzyme in the degradation pathway of nonylphenol and bisphenol A (Kolvenbach et al. 2007; Gabriel et al. 2007a; Gabriel et al. 2007b; Gabriel et al. 2005; Corvini et al. 2006), the HQDO represents an interesting class of enzymes that has been little studied.

The enzyme readily lost activity upon exposure to its substrate HQ, which distinguishes it from HQDO from *Pseudomonas fluorescens *ACB (Moonen et al. 2008b). This characteristic has previously also been reported for a HQDO from a *Moraxella *strain(Spain and Gibson 1991), and for other extradiol type dioxygenases, such as catechol dioxygenases (Cerdan et al. 1994; Bartels et al. 1984) and protocatechuate dioxygenases (Ono et al. 1970). This is possibly due to oxidation of ferrous iron in the active centre to ferric iron, a process that is reversed *in vivo *by redox-dependent reactions catalyzed by ferredoxins (Tropel et al. 2002).

The hypothesis of a ferrous iron in the active center of the enzyme is strongly supported by the results from experiments with hydrogen peroxide or with chelators of ferrous iron, in which enzyme activity was significantly reduced, Hydrogen peroxide is likely to inactivate the enzyme by oxidizing the ferrous iron to its ferric form (Spain and Gibson 1991; Lendenmann and Spain 1996; Moonen et al. 2008b).

The molecular mass determined by size exclusion chromatography in combination with the molecular masses determined by SDS-PAGE and the similarities to HQDO of *Pseudomonas fluorescens *ACB (Moonen et al. 2008b) indicate that the enzyme may be a tetramer in its native form. The iron content determined by ICP-MS suggests the presence of 1.4 atoms of iron per tetrameric unit. Taking into account that the loss of activity during the purification process may partially have been caused by the removal of iron from the enzyme, it can be assumed that the actual enzyme contains two iron atoms per tetrameric unit. This would be in agreement with data reported for other heteromeric extradiol dioxygenases, namely the protocatechuate 4,5-dioxygenases from *Pseudomonas pseudoalcaligenes *JS45 (Lendenmann and Spain 1996) and *Sphingomonas paucimobilis *SYK-6 (Sugimoto et al. 1999).

Our findings support reports that the substituent in *para *to the hydroxyl group adjacently to the cleaving site is an important discriminator for substrate binding to HQDO (Moonen et al. 2008b). Phenolic compounds possessing functional groups in *para *to the hydroxyl group, i.e. 4-mercaptophenol, 4-hydroxybenzonitrile and 4-nitrophenol, exhibited a strong inhibitory effect, whereas those lacking substituents in *para *position, such as phenol, catechol and resorcinol, led to enzyme inhibition of less than 10%. Furthermore, less than 5% inhibition was observed with caffeic acid and *p*-coumaric acid, which might indicate that the propenyl side chain in para position is sterically preventing the inhibitor from accessing the active site of the enzyme. The strong inhibitory effect on HQDO observed benzoquinone can be of relevance *in vivo*, as benzoquinone may be formed in the cell by oxidation of hydroquinone.

The degradation of technical nonylphenol mixtures in *Sphingomonas *sp. TTNP3 and *Sphingobium xenophagum *Bayram leads to the formation of minor amounts of 2-alkylated hydroquinones (Corvini et al. 2004a; Gabriel et al. 2005), potentially toxic metabolites that may pose oxidative stress on the organism. Even though *Sphingomonas *sp. TTNP3 has shown to lack the ability to degrade both 2(3',5'-dimethyl-3'-heptyl)-1,4-benzenediol and 2(2',6'-dimethyl-2'-heptyl)-1,4-benzenediol (Corvini et al. 2006) we wanted to investigate if other alkylated hydroquinones could be degraded by HQDO. Interestingly, degradation of 2-methylhydroquinone appeared to proceed at a higher rate than that of HQ, whereas with increasing length of the alkyl chain degradation (e.g. 2-hexylhydroquinone) rates decreased to less than two per cent of the degradation rate of HQ.

2-methylhydroquinone seemed to be preferentially cleaved adjacently to a ring-hydrogen, and not the electron donating methyl substituent. In contrast, type I HQDO in *Sphingomonas paucimobilis *(LinE) and type II HQDO in *Pseudomonas fluorescens *ACB appear to cleave 2-chlorohydroquinone and 2-fluorohydroquinone, respectively, both between two carbon atoms (C-1 and C-2) substituted by a hydroxyl and the electron withdrawing halogen group (Miyauchi et al. 1999; Moonen et al. 2008b). Future degradation experiments with a halogen monosubstituted hydroquinone derivative will determine whether steric or electronic constraints are predominant in determining the cleavage site.

Interestingly, 2-t-butylhydroquinone appeared to be degraded slower than linear 2-propyl- and 2-pentylhydroquinone, respectively, although a degradation rate in the same range as that of the latter derivatives would have been expected. A direct comparison to 2-butylhydroquinone, which bears a linear alkyl, was not possible as it could not be commercially obtained. Concluding from our data, an involvement of HQDO in the degradation of 2-nonylhydroquinones appears improbable, as the apparent degradation of 2-(1-methyl-1-octyl)hydroquinone was not unequivocally distinguishable from the oxygen consumption caused by chemical oxidation of the substrate. But considering the apparent negative effects of both alkyl chain length and the occurrence of branched chains it can be reasoned that other 2-nonylhydroquinone isomers, branched or not, will not be degraded by HQDO.

Quinonoide compounds, derived from hydroquinones are agents of oxidative stress and have a high toxic potential (Monks et al. 1992; Kappus 1987), which is why a rapid further metabolization of this intermediate is necessary to minimize exposure time and thus to avoid damage to the cell. Therefore, the elucidation of the nature of HQDO contributes substantially to the understanding of the mechanisms to prevent oxidative stress present in *Sphingomonas *sp. strain TTNP3.

According to our results the deduced amino acid sequences of the subunits of HQDO of *Sphingomonas *sp. TTNP3 show similarities to sequences of HQDO and putative proteins found in other strains, namely *Photorhabdus*, *Pseudomonas*, *Burkholderia*, and *Variovorax *(Figure 4). No sequence similarities to known sequences of HQDO reported for other sphingomonads could be found. Surprisingly, both HqdA and HqdB were found to be most similar to homologous proteins from *Photorhabdus luminescens *subsp. *laumondii *TTO1, a bacterium that can be found in the gut of entomopathogenic nematodes (Bowen et al. 1998).

Our data show that the HQDO of strain TTNP3, can be attributed to the type II HQDO. It represents the first enzyme of this type that has been identified in a *Sphingomonas *strain.

## Competing interests

The authors declare that they have no competing interests.

## Authors' contributions

BAK carried out the enzyme purification and biochemical experiments and drafted the manuscript. ML performed IPC-MS analyses. DB and ER carried out protein analysis and identification. JF and CV performed the genome sequencing and assembly and provided nucleotide sequence data. FLPG elaborated GC-MS data, conceived fragmentation patterns and commented on the manuscript. HPEK participated in the design of the study and commented on the manuscript. AS and PFXC participated in the design of the study, commented on the manuscript and supervised the Ph.D. thesis of BAK from which large parts of this study originated.

## Supplementary Material

Additional file 1 **Proposed fragmentation pattern for trimethylsilylated 4-hydroxymuconic semialdehyde and its methylated analogon**. 1, proposed GC-MS fragmentation pattern of 4-hydroxymuconic semialdehyde; 2, proposed GC-MS fragmentation pattern of the ring-cleavage product of 2-methyl-hydroquinone with cleavage sites between the ring bonds C(1)-C(6), C(3)-C(4) and C(5)-C(6), respectively; 3, proposed GC-MS fragmentation pattern of the ring-cleavage product of 2-methyl-hydroquinone with cleavage sites between the ring bonds C(1)-C(2); 4, GC-MS fragmentation pattern of 2-hydroxy-6-(2-hydroxyphenyl)-6oxo-2,4-hexadienoic acid (Kohler et al. 1993). Click here for file

Additional file 2 **Multiple sequence alignment performed by ClustalW 2 of the sequence of HqdA with sequences retrieved by BLAST search**. Shown is the original multiple sequence alignment from which Figure 4A has been rendered. Click here for file

Additional file 3 **Multiple sequence alignment performed by ClustalW 2 of the sequence of HqdB with sequences retrieved by BLAST search**. Shown is the original multiple sequence alignment from which Figure 4B has been rendered. Click here for file

## References

[B1] Bartels I Knackmuss H-J Reineke W Suicide Inactivation of Catechol 2,3-Dioxygenase from Pseudomonas putida mt-2 by 3-Halocatechols Appl Environ Microbiol 1984 47 3 500 505 16346490 10.1128/aem.47.3.500-505.1984 PMC239710

[B2] Bowen D Rocheleau TA Blackburn M Andreev O Golubeva E Bhartia R ffrench-Constant RH Insecticidal Toxins from the Bacterium Photorhabdus luminescens Science 1998 280 5372 2129 2132 10.1126/science.280.5372.2129 9641921

[B3] Cai M Xun L Organization and Regulation of Pentachlorophenol-Degrading Genes in Sphingobium chlorophenolicum ATCC 39723 J Bacteriol 2002 184 17 4672 4680 10.1128/JB.184.17.4672-4680.2002 12169590 PMC135293

[B4] Cerdan P Wasserfallen A Rekik M Timmis KN Harayama S substrate-specificity of catechol 2,3-dioxygenase encoded by tol plasmid pwwo of pseudomonas-putida and its relationship to cell-growth Journal of Bacteriology 1994 176 19 6074 6081 7928969 10.1128/jb.176.19.6074-6081.1994 PMC196827

[B5] Chauhan A Samanta SK Jain RK Degradation of 4-nitrocatechol by Burkholderia cepacia: a plasmid-encoded novel pathway Journal of Applied Microbiology 2000 88 5 764 772 10.1046/j.1365-2672.2000.01018.x 10792536

[B6] Corvini PFX Hollender J Ji R Schumacher S Prell J Hommes G Priefer U Vinken R Schaffer A The degradation of alpha-quaternary nonylphenol isomers by Sphingomonas sp strain TTNP3 involves a type II ipso-substitution mechanism Applied Microbiology and Biotechnology 2006 70 1 114 122 10.1007/s00253-005-0080-0 16091931

[B7] Corvini PFX Meesters RJW Schaffer A Schroder HF Vinken R Hollender J Degradation of a nonylphenol single isomer by Sphingomonas sp strain TTNP3 leads to a hydroxylation-induced migration product Applied and Environmental Microbiology 2004 70 11 6897 6900 10.1128/AEM.70.11.6897-6900.2004 15528560 PMC525215

[B8] Corvini PFX Meesters RJW Schäffer A Schröder HF Vinken R Hollender J Degradation of a nonylphenol single isomer by Sphingomonas sp. strain TTNP3 leads to a hydroxylation-induced migration product Applied and Environmental Microbiology 2004 70 11 6897 6900 10.1128/AEM.70.11.6897-6900.2004 15528560 PMC525215

[B9] Corvini PFX Vinken R Hommes G Schmidt B Dohmann M Degradation of the radioactive and non-labelled branched 4(3 ',5 '-dimethyl 3 '-heptyl)-phenol nonylphenol isomer by Sphingomonas TTNP3 Biodegradation 2004 15 1 9 18 14971853 10.1023/b:biod.0000009937.20251.d2

[B10] Dogra C Raina V Pal R Suar M Lal S Gartemann K-H Holliger C van der Meer JR Lal R Organization of lin Genes and IS6100 among Different Strains of Hexachlorocyclohexane-Degrading Sphingomonas paucimobilis: Evidence for Horizontal Gene Transfer J Bacteriol 2004 186 8 2225 2235 10.1128/JB.186.8.2225-2235.2004 15060023 PMC412113

[B11] Eltis LD Bolin JT Evolutionary relationships among extradiol dioxygenases Journal of Bacteriology 1996 178 20 5930 5937 8830689 10.1128/jb.178.20.5930-5937.1996 PMC178449

[B12] Eppink MHM Cammaart E van Wassenaar D Middelhoven WJ van Berkel WJH Purification and properties of hydroquinone hydroxylase, a FAD-dependent monooxygenase involved in the catabolism of 4-hydroxybenzoate in Candida parapsilosis CBS604 European Journal of Biochemistry 2000 267 23 6832 6840 10.1046/j.1432-1033.2000.01783.x 11082194

[B13] Ferraroni M Seifert J Travkin VM Thiel M Kaschabek S Scozzafava A Golovleva L Schlomann M Briganti F Crystal structure of the hydroxyquinol 1,2-dioxygenase from Nocardioides simplex 3E, a key enzyme involved in polychlorinated aromatics biodegradation Journal of Biological Chemistry 2005 280 22 21144 21154 10.1074/jbc.M500666200 15772073

[B14] Gabriel FLP Cyris M Giger W Kohler HPE ipso-substitution: A general biochemical and biodegradation mechanism to cleave alpha-quaternary alkylphenols and bisphenol A Chemistry & Biodiversity 2007 4 9 2123 2137 10.1002/cbdv.200790170 17886831

[B15] Gabriel FLP Cyris M Jonkers N Giger W Guenther K Kohler HPE Elucidation of the ipso-substitution mechanism for side-chain cleavage of alpha-quaternary 4-nonylphenols and 4-t-butoxyphenol in Sphingobium xenophagum Bayram Applied and Environmental Microbiology 2007 73 10 3320 3326 10.1128/AEM.02994-06 17369338 PMC1907130

[B16] Gabriel FLP Heidlberger A Rentsch D Giger W Guenther K Kohler HPE A novel metabolic pathway for degradation of 4-nonylphenol environmental contaminants by Sphingomonas xenophaga Bayram-ipso-hydroxylation and intramolecular rearrangement* Journal of Biological Chemistry 2005 280 16 15526 15533 10.1074/jbc.M413446200 15665329

[B17] Jain RK Dreisbach JH Spain JC Biodegradation of p-nitrophenol via 1, 2, 4-benzenetriol by an Arthrobacter sp Applied and Environmental Microbiology 1994 60 8 3030 3032 8085840 10.1128/aem.60.8.3030-3032.1994 PMC201761

[B18] Kappus H Oxidative stress in chemical toxicity Archives of Toxicology 1987 60 1 144 149 10.1007/BF00296968 3304204

[B19] Kohler HPE Schmid A van der Maarel M Metabolism of 2, 2'-dihydroxybiphenyl by Pseudomonas sp. strain HBP1: production and consumption of 2, 2', 3-trihydroxybiphenyl Journal of Bacteriology 1993 175 6 1621 1628 8449871 10.1128/jb.175.6.1621-1628.1993 PMC203955

[B20] Kolvenbach B Schlaich N Raoui Z Prell J Zuhlke S Schaffer A Guengerich FP Corvini PFX Degradation Pathway of Bisphenol A: Does ipso Substitution Apply to Phenols Containing a Quaternary {alpha}-Carbon Structure in the para Position? Appl Environ Microbiol 2007 73 15 4776 4784 10.1128/AEM.00329-07 17557840 PMC1951029

[B21] Laemmli UK Cleavage Of Structural Proteins During Assembly Of Head Of Bacteriophage-T4 Nature 1970 227 5259 680. 10.1038/227680a0 5432063

[B22] Lal R Pandey G Sharma P Kumari K Malhotra S Pandey R Raina V Kohler HPE Holliger C Jackson C Oakeshott JG Biochemistry of microbial degradation of hexachlorocyclohexane and prospects for bioremediation Microbiology and Molecular Biology Reviews 2010 74 1 58 80 10.1128/MMBR.00029-09 20197499 PMC2832351

[B23] Larkin MA Blackshields G Brown NP Chenna R McGettigan PA McWilliam H Valentin F Wallace IM Wilm A Lopez R Thompson JD Gibson TJ Higgins DG Clustal W and Clustal × version 2.0 Bioinformatics 2007 23 21 2947 2948 10.1093/bioinformatics/btm404 17846036

[B24] Latus M Seitz HJ Eberspacher J Lingens F Purification and characterization of hydroxyquinol 1, 2-dioxygenase from Azotobacter sp. strain GP1 Applied and Environmental Microbiology 1995 61 7 2453 2460 16535063 10.1128/aem.61.7.2453-2460.1995 PMC1388481

[B25] Lendenmann U Spain J 2-aminophenol 1,6-dioxygenase: a novel aromatic ring cleavage enzyme purified from Pseudomonas pseudoalcaligenes JS45 J Bacteriol 1996 178 21 6227 6232 8892823 10.1128/jb.178.21.6227-6232.1996 PMC178494

[B26] Machonkin T Holland P Smith K Liberman J Dinescu A Cundari T Rocks S Determination of the active site of Sphingobium chlorophenolicum 2,6-dichlorohydroquinone dioxygenase (PcpA) Journal of Biological Inorganic Chemistry 2009 15 3 291 301 10.1007/s00775-009-0602-9 19924449

[B27] Manickam N Reddy MK Saini HS Shanker R Isolation of hexachlorocyclohexane-degrading *Sphingomonas *sp. by dehalogenase assay and characterization of genes involved in Y-HCH degradation Journal of Applied Microbiology 2008 104 4 952 960 10.1111/j.1365-2672.2007.03610.x 18042212

[B28] Miyauchi K Adachi Y Nagata Y Takagi M Cloning and sequencing of a novel meta-cleavage dioxygenase gene whose product is involved in degradation of gamma-hexachlorocyclohexane in Sphingomonas paucimobilis Journal of Bacteriology 1999 181 21 6712 6719 10542173 10.1128/jb.181.21.6712-6719.1999 PMC94136

[B29] Monks TJ Hanzlik RP Cohen GM Ross D Graham DG Quinone chemistry and toxicity Toxicology and Applied Pharmacology 1992 112 1 2 16 10.1016/0041-008X(92)90273-U 1733045

[B30] Moonen MJH Kamerbeek NM Westphal AH Boeren SA Janssen DB Fraaije MW van Berkel WJH Elucidation of the 4-hydroxyacetophenone catabolic pathway in Pseudomonas fluorescens ACB Journal of Bacteriology 2008 190 15 5190 5198 10.1128/JB.01944-07 18502868 PMC2493259

[B31] Moonen MJH Synowsky SA van den Berg WAM Westphal AH Heck AJR van den Heuvel RHH Fraaije MW van Berkel WJH Hydroquinone dioxygenase from Pseudomonas fluorescens ACB: a novel member of the family of nonheme-iron(II)-dependent dioxygenases Journal of Bacteriology 2008 190 15 5199 5209 10.1128/JB.01945-07 18502867 PMC2493252

[B32] Ono K Nozaki M Hayaishi O Purification and some properties of protocatechuate 4,5-dioxygenase Biochimica et Biophysica Acta (BBA)-Enzymology 1970 220 2 224 238 10.1016/0005-2744(70)90008-2 5487881

[B33] Perkins DN Pappin DJC Creasy DM Cottrell JS Probability-based protein identification by searching sequence databases using mass spectrometry data Electrophoresis 1999 20 18 3551 3567 10.1002/(SICI)1522-2683(19991201)20:18<3551::AID-ELPS3551>3.0.CO;2-2 10612281

[B34] Rieble S Joshi DK Gold MH Purification and characterization of a 1, 2, 4-trihydroxybenzene 1, 2-dioxygenase from the basidiomycete Phanerochaete chrysosporium Journal of Bacteriology 1994 176 16 4838 4844 8050996 10.1128/jb.176.16.4838-4844.1994 PMC196317

[B35] Shen W Liu W Zhang J Tao J Deng H Cao H Cui Z Cloning and characterization of a gene cluster involved in the catabolism of p-nitrophenol from Pseudomonas putida DLL-E4 Bioresource Technology 2010 in press 10.1016/j.biortech.2010.04.052 20466541

[B36] Spain JC Gibson DT Pathway For Biodegradation Of Para-Nitrophenol In A Moraxella Sp Applied and Environmental Microbiology 1991 57 3 812 819 16348446 10.1128/aem.57.3.812-819.1991 PMC182799

[B37] Sugimoto K Senda T Aoshima H Masai E Fukuda M Mitsui Y Crystal structure of an aromatic ring opening dioxygenase LigAB, a protocatechuate 4,5-dioxygenase, under aerobic conditions Structure 1999 7 8 953 965 10.1016/S0969-2126(99)80122-1 10467151

[B38] Takenaka S Okugawa S Kadowaki M Murakami S Aoki K The metabolic pathway of 4-aminophenol in Burkholderia sp strain AK-5 differs from that of aniline and aniline with C-4 substituents Applied and Environmental Microbiology 2003 69 9 5410 5413 10.1128/AEM.69.9.5410-5413.2003 12957929 PMC194951

[B39] Travkin VM Jadan AP Briganti F Scozzafava A Golovleva LA Characterization of an intradiol dioxygenase involved in the biodegradation of the chlorophenoxy herbicides 2,4-D and 2,4,5-T FEBS Lett 1997 407 1 69 72 10.1016/S0014-5793(97)00297-4 9141483

[B40] Tropel D Meyer C Armengaud J Jouanneau Y Ferredoxin-mediated reactivation of the chlorocatechol 2,3-dioxygenase from Pseudomonas putida GJ31 Archives of Microbiology 2002 177 4 345 351 10.1007/s00203-002-0399-1 11889489

[B41] Vester D Rapp E Gade D Genzel Y Reichl U Quantitative analysis of cellular proteome alterations in human influenza A virus-infected mammalian cell lines Proteomics 2009 9 12 3316 3327 10.1002/pmic.200800893 19504497

[B42] Wei Q Liu H Zhang J-J Wang S-H Xiao Y Zhou N-Y Characterization of a para-nitrophenol catabolic cluster in Pseudomonas sp. strain NyZ402 and construction of an engineered strain capable of simultaneously mineralizing both para- and ortho-nitrophenols Biodegradation 2010 21 6 915 921 10.1007/s10532-010-9351-2 20049512

[B43] Xu L Resing K Lawson SL Babbitt PC Copley SD Evidence That pcpA Encodes 2,6-Dichlorohydroquinone Dioxygenase, the Ring Cleavage Enzyme Required for Pentachlorophenol Degradation in Sphingomonas chlorophenolica Strain ATCC 39723 Biochemistry 1999 38 24 7659 7669 10.1021/bi990103y 10387005

[B44] Yamamoto S Otsuka S Murakami Y Nishiyama M Senoo K Genetic diversity of gamma-hexachlorocyclohexane-degrading sphingomonads isolated from a single experimental field Letters in Applied Microbiology 2009 49 4 472 477 10.1111/j.1472-765X.2009.02691.x 19674290

[B45] Yin Y Zhou N-Y Characterization of MnpC, a Hydroquinone Dioxygenase Likely Involved in the meta-Nitrophenol Degradation by Cupriavidus necator JMP134 Current Microbiology 2010 61 5 471 476 10.1007/s00284-010-9640-3 20386911

[B46] Zhang J-J Liu H Xiao Y Zhang X-E Zhou N-Y Identification and Characterization of Catabolic para-Nitrophenol 4-Monooxygenase and para-Benzoquinone Reductase from Pseudomonas sp. Strain WBC-3 J Bacteriol 2009 191 8 2703 2710 10.1128/JB.01566-08 19218392 PMC2668391

