# Supplementary material for: Purification and characterization of hydroquinone dioxygenase from Sphingomonas sp. strain TTNP3
Source: AMB Express. 2011 May 27;1:8. doi: 10.1186/2191-0855-1-8 (PMC3222310; doi:10.1186/2191-0855-1-8)

CLUSTAL 2.0.11 MULTIPLE SEQUENCE ALIGNMENT

File: F:/HqdA sequence alignment.ps **Date: Fri Apr 01 15:23:07 2011**


```
                                         * *.::  : :* ::     .  .*.*** :::*  : **:::. . *  *.:*  *::* * *::.:**** : :* .:.:.:: *            *:       * *: ** : * .** ***   **:: :   . .
Burkholderia_ambifaria_IOP40-1 -MTDTAFHTVFGSLDSYTKGEIEITSGSARHYAFSNVFEVASKSAPYEKVVAGKNLEYVIEVLRTDGESPWFACAHDEFAIQMDGEVRIEFIKLDNPPTTGR----GTVSA-GTQPAGREMGYVVLRKGHQALLPAGCAYRFTAD-KPGV  143
Burkholderia_ambifaria_MC40-6  -MTDTAFHTVFGSLDSYTKGEIEITSGSAQHYAFSNVFEVASKSAPYEKVVAGKNLEYVIEVLRTDGESPWFACAHDEFAIQMDGDVRIEFIKLDNPPTTGR----GTVSA-GTQPAGREMGYVVLRKGHQALLPAGCAYRFAAD-KPGV  143
Burkholderia_ambifaria_AMMD    -MTDTAFHTVFGSLDSYTKGEIEITSGSAQHYAFSNVFEVASKSAPYEKVVAGKNLEYVIEVLRTDGQSPWFACAHDEFAIQMDGEVRIEFIKLDNPPTTGR----GTVSA-GTQPAGREMGYVVLRKGHQALLPAGCAYRFSAD-QPGV  143
Burkholderia_ambifaria_MEX-5   -MTDTAFHTVFGSLDSYTKGEIEITSGSAQHYAFSNVFEVASKSAPYEKVVAGKNLEYVIEVLRTDGQSPWFACTHDEFAIQMDGEVRIEFIKLDNPPTSSR----GTVSA-GTQPAGREMGYVVLRKGHQALLPAGCAYRFSAV-KPGV  143
Burkholderia_multivorans_CGD2M -MTDTARHTVFGSLDSYTRGEIEITSGSAKHYAFSNIFDIASKSAPYEKVVAGKNLEYVIEVLRTEGESPWFACAHDEFAIQMDGEVRIDFIKLDSPPTTGR----GTVSA-GVEPAGREMGHVVLRKGHQALLPAGCAYRFIAS-KPGV  143
Burkholderia_multivorans_CGD1  -MTDTARHTVFGSLDSYTRGEIEITSGSAKHYAFSNIFDIASKSAPYEKVVAGKNLEYVIEVLRTEGESPWFACAHDEFAIQMDGEVRIEFIRLDSPPTTGR----GTVSA-GIRPAGREMGHVVLRKGHQALLPAGCAYRLIAS-KPGV  143
Burkholderia_multivorans_ATCC_ -MTDTARHTVFGSLDSYTRGEIEITSGSAKHYAFSNIFDIASKSAPYEKVVAGKNLEYVIEVLRTEGESPWFACAHDEFAIQMDGEVRIEFIKLHSPPTTGR----GTVSA-GIQPAGREMGYVVLRKGHQALLPAGCAYRFIAS-KPGV  143
Burkholderia_cenocepacia_HI242 -MTSTAFHTVFGSLDGYRKGEIEITSGSAQHYAFSNVFEVASKSAPYEKVVAGKNLEYVIEVLRTDGQSPWFACAHDEFAIQMDGEVRIDFIKLDDPPQSGR----GTVRA-GAHPAGRNMGYVVLRKGHQALLPAGCAYRFTAS-RPGV  143
Burkholderia_cenocepacia_J2315 -MTSTAFHTVFGSLDGYRKGEIEITSGSAQHYAFSNVFEVASKSAPYEKVVAGKNLEYVIEVLRTDGQSPWFACAHDEFTIQMDGEVRIDFIKLDHPPQAGR----GTVSA-GAHPAGRNMGYVVLRNGHQALLPAGCAYRFTAS-RPGV  143
Burkholderia_sp._383           -MTDTAFHTVFGSLDHYRKGEIEITSGSAQHYAFSNVFEVASKSAPYEKVVAGKNLEYVIEVLRTDGQSPWFACSHDEFVIQMDGEVRIEFIKLDTPPQSAR----GTVSA-GMQPAGRKMGYVVLRQGHQALLPAGCAYRFTAS-QPGV  143
Burkholderia_sp._CCGE1002      -MTESSFHTVFGSLDGYRKGEIEITSGDARHYVFSNIFEVASESAPYQKVVVGKNLDYVIETLRTEGRSQWFACAHDEFAILMDGEVRIDFIKLDNPPKNGT----GTVAA-GAQPAGRAMGHVVLRRGHQALLPAGCAYRFTAQ-KSGV  143
Burkholderia_sp._H160          -MTEQSFHTVFGSLDGYRKGEIEITSGEARHYVFSNIFEVASEAKPYEKVVVGKNLDYVIEALRTEGQSPWFACAHDEFTIQMDGEVRIDFIKLDNPPKSGT----GTVVA-GAQPAGRAMGHVVLRRGHQALLPAGCAYRFTAH-KTGV  143
Pseudomonas_sp._1-7            -MSNAAVNTVFASLDNFRKGTVEIISGEARHYAFSNIFEVAQNSKPYEKVVVGLNLGYVVETLRAEGQSPWFTAAHDEFAIVMDGEVRVEFLKLDAPSKHGE----GTHLA-GELPVGKPMGYVLLKRGHQCLLPAGSAYRFEAS-RPGV  143
Pseudomonas_sp._WBC-3          -MSNVAVNTVFASLDNFRKGTVEIISGEARHYAFSNIFEVAQNSKPYEKVVVGLNLGYVVETLRAEGQSPWFTAAHDEFAIVMDGEVRVEFLKLDAPSKHGE----GTHLA-GELPVGKPMGYVLLKRGHQCLLPAGSAYRFEAS-RPGV  143
Pseudomonas_putida             -MSTAAIKTVFGSLENYTKGTVEIISGEARHYAFSNIFEVAEKSPPYQKVVVGLNLGYVIETLRAEGQSPWFTAAHDEFAIVMDGEVRVDFLKLDTPLAEGE----GTRLA-GEIPAGKPMGYVLLKRGHQCLLPAGTAYRFESS-RPGV  143
Pseudomonas_sp._NyZ402         -MSTAAIKTVFGSLENYTKGSVELISGEARHYAFSNIFEVAENSPPYQKVVVGLNLGYVIETLRTEGQSPWFTASHDEFAIVMDGEVRVDFLKLDTPLAEGE----GTRLA-GEIPVGKPMGYVLLKRGHQCLLPAGTAYRFESS-RPGV  143
Pseudomonas_aeruginosa_PA7     --MNAAIRTVFGSLGDYRKGSIELVKGDARHYAFSNVFEVAARSAPYEKVVVGLNLGYVIEALRAEGRSPWFACSHDEFAISMDGEVRVDFLDLERPPREGE----GTQLA-GDLPRGRPMGYVLLGRGHQVLLPAGRAYRFEAA-RPCV  142
Pseudomonas_fluorescens        MSTQPAFKTVFGSLAQYTKGSIELVTGKKAHYAFSNIFEVAAKSAPYEKVVVGANLGYVIETLRAEGTSPWFACSHDEFLISVDGSVRIDFLKLDETPLAGD----GTRLA-GELPKGKPMGYVLLKRGHQALLPAGCAYRFEAS-NPCV  144
Variovorax_paradoxus_S110      --MTVAYQTRFGSLKHFEKGHVEPIDDDVRHYAFSNCFEIASISRPYEKVVFGQNQIYVLETLRAEGTSPWYTSAHDEFALVMDGEVEVHLVQLDAAQAVADKDKNGAVLV-QGEPRGRKMGWMKLSRGHQGLLPKNTAYQFRAAGDPGV  147
Photorhabdus_luminescens_subsp --MLVQYKTAFASIDNYHKGGIQALVEDPKRYVFSNMYEIAATSAPYDRIVAAINLDYTIEIARAEGTSPWFACAHDEFVVAMDYDVEVHFVKLTD-ESVIDEDKDGAVKL-NGTPDGQKMGCIYLKRGHQALLPEKVAYRFHAD-KPAT  145
Sphingomonas_sp._strain_TTNP3  ---MADVVTEFGALTDYRKGGVEIIDDDPRNYVFSNVFEVAANAAPYERVAVGKNFEYVIESARAEGTSGWFSCAHDEFVLAMDGQIEVHLLKLDNSDAYVDPDSEGAVAIGEALPEGRKMGRIVLRRGHMALLPVGAAYRFYAE-QPAA  146
                               1.......10........20........30........40........50........60........70........80........90.......100.......110.......120.......130.......140.......150
```

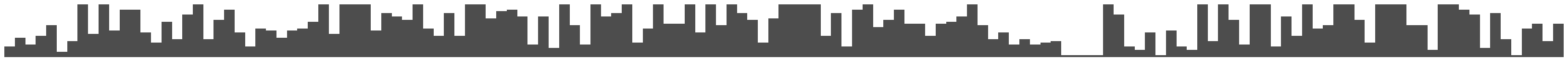

```
                                : *:  * ::*:**.:**
Burkholderia_ambifaria_IOP40-1 ALVQTVLGELSVEKWADICLH---  164
Burkholderia_ambifaria_MC40-6  ALVQTVLGELSVEKWADICLP---  164
Burkholderia_ambifaria_AMMD    ALVQTVLGELSVEKWADICLH---  164
Burkholderia_ambifaria_MEX-5   ALVQTVLGELSVEKWADICLH---  164
Burkholderia_multivorans_CGD2M ALVQTVLGELSVEKWAEICVH---  164
Burkholderia_multivorans_CGD1  ALVQTVLGELSVEKWAEICVH---  164
Burkholderia_multivorans_ATCC_ ALVQTVLGELSVEKWAEICVH---  164
Burkholderia_cenocepacia_HI242 ALVQTVLGELSVEKWAEICLH---  164
Burkholderia_cenocepacia_J2315 ALVQTVLGELSVEKWAEICLH---  164
Burkholderia_sp._383           ALVQTVLGELSVEKWADICLH---  164
Burkholderia_sp._CCGE1002      VLVQTMLGELSVEKWADICLH---  164
Burkholderia_sp._H160          ALVQTMLGELSVEKWADICLH---  164
Pseudomonas_sp._1-7            ILQQTIKGPLSVEKWAEICLK---  164
Pseudomonas_sp._WBC-3          ILQQTIKGPLSVEKWAEICLK---  164
Pseudomonas_putida             LLQQTIKGPLSVEKWAEICLK---  164
Pseudomonas_sp._NyZ402         LLQQTIKGPLSVEKWADICLK---  164
Pseudomonas_aeruginosa_PA7     LLQQTILGPLSVEKWADICLK---  163
Pseudomonas_fluorescens        LLQQTVLGPLSVEKWADICLK---  165
Variovorax_paradoxus_S110      IVLQTCKGDLSVEKWADICQTQ--  169
Photorhabdus_luminescens_subsp LMIQSILGDVSVQKWADICLN---  166
Sphingomonas_sp._strain_TTNP3  MLFQSIEGAVTVQKWGEICQTEAA  170
                               .......160.......170....
```

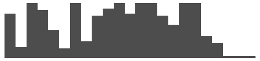

Supplement: Additional file 2 — Multiple sequence alignment performed by ClustalW 2 of the sequence of HqdA with sequences retrieved by BLAST search. Shown is the original multiple sequence alignment from which Figure 4A has been rendered. [file 2191-0855-1-8-S2.PDF]
