# Supplementary material for: Purification and characterization of hydroquinone dioxygenase from Sphingomonas sp. strain TTNP3
Source: AMB Express. 2011 May 27;1:8. doi: 10.1186/2191-0855-1-8 (PMC3222310; doi:10.1186/2191-0855-1-8)

CLUSTAL 2.0.11 MULTIPLE SEQUENCE ALIGNMENT

File: F:/hqdB sequence alignment.ps **Date: Fri Apr 01 15:19:56 2011**


```
                                                         .   **: :  * * : * ***. :           * :  . :*:::***:******* * ** *:** *:*..* :: * *:     ** :  * * :  : * *: :: **.   :*****
Pseudomonas_sp._1-7            MAMLESAV---DSAAFADDDVKASPPHAVTGYRSFQLGAFELSRDEYFARITWPAK-GETRSHLIPADIFLRALMRDVAWGFFYGWVNFDHVIGTRNYYGKVDLYAGTFNGTLKAAGVNYTENFETPLIMATFKAILRDWTNATFDPFAA  146
Pseudomonas_sp._WBC-3          MAMLESAV---DSAAFADDDVQASPPHAVTGYRSFQLGAFELSRDEYFARITWPAK-GETRSHLIPADIFLRAMMRDVAWGFFYGWVNFDHVIGTRNYYGKVDLYAGTFNGTLKAAGVNYTENFETPLIMATFKAILRDWTNATFDPFAA  146
Pseudomonas_putida             MAMLESAV---EAPSFADDEVNASAPHPITGYRSFRLGAFELSRDEYFARITWPAK-GETRSHLIPADIFLRALMRDVAWGFFYGWVNFDSVIGTRNYYGKVDLYAGTFNGVLKEAGVNYTENFETPLIMATFKAMLRDWVNCTFDPFAA  146
Pseudomonas_sp._NyZ402         MAMLETVV---EAPSFADDEVNASAPHPVTGYRSYRLGAFELSRDEYFARITWPAK-GETRSHLIPADIFLRALMRDVAWGFFYGWVNFDNVIGTRNYYGKVDLYAGTFNGVLKEAGVNYTENFETPLIMATFKAMLRDWVNCTFDPFAA  146
Pseudomonas_fluorescens_ACB    MAMLEAVETE-NALSFADDLVTASAPHLVTGYKAFELGSFNLSRDEYFARIEWPAK-GEQRSHLIPADAFLRSVMRDVAWGFFYGWVNFDHVFGTRNHYGKVDMYAGTFNGVLKAAGVDYTEQFETPLIMATFKAILRDWVNAGFDPFAA  148
Pseudomonas_aeruginosa_PA7     MAMLDTPE---QTRGFASDEVEAGRPDPATGYRSFRLGAFTLSRDEYFARVEWPAK-GQSRSHLIPVDAFLRAMMRDVAWGFFYGWVNFDHVIGTRNHYGKVDLYAGTFNGVLREAGVDYTERFDTPKIMATFKAVLRDWTNASFDPFAA  146
Burkholderia_sp._CCGE1002      MASLETLD---HPAGFAADTVRASEPDAVTGYRRFTLGAFEFLRDEYFVKINWPAR-GQTRTHAVPADAFLRAMMRDVAWGFFYGWVNFDHVFGTRNHYGKVDVYAGSFNGIMKDAGVDYMETFETPTIMATFKAILHDWTNEGFDPFAA  146
Burkholderia_sp._H160          MASLETLD---HPAGFAADTVRASEPDAITGYRRFTLGAFEFLRDEYFVKINWPAK-GQTRTHAVPADAFLRAMMRDVAWGFFYGWVNFDHVFGTRNHYGKVDMYAGSFNGIMKDAGVDYMETFETPTIMATFKAILHDWTNEGFDPFAA  146
Burkholderia_cenocepacia_HI242 MATLETLD---LSTGFAADLVRSTEADAVTGYRRFQLGEFAFQRDEYFVKISWPAK-GQTRTHAMPADAFLRAMMRDVAWGFFYGWVNFDHVFGTRNHYGKVDMYAGTFNGILKDAGVDYTETFDTPTIMATFKAMLHDWTNEGFDPFAA  146
Burkholderia_cenocepacia_J2315 MATLETLD---LSTGFAADLVRSTEADAVTGYRRFQLGEFAFQRDEYFVKISWPAK-GQTRTHAMPADAFLRAMMRDVAWGFFYGWVNFDHVFGTRNHYGKVDMYAGTFNGILKDAGVDYTETFDTPTIMATFKAMLHDWTNEGFDPFAA  146
Burkholderia_multivorans_CGD2M MATLETLD---LSAGFAADLVQVTQADAVTGYRRFQLGEFAFQRDEYFVKISWPAR-GQTRTHAIPADAFLRAMMRDVAWGFFYGWVNFDHVFGTRNHYGKVDVYAGTFNGILKEAGVDYTETFETPTVMATFKAMLHDWTNEGFDPFAA  146
Burkholderia_multivorans_CGD1  MATLETLD---LSAGFAADLVQATQANAVTGYRRFQLGEFAFQRDEYFVKISWPAK-GQTRTHAIPADAFLRAMMRDVAWGFFYGWVNFDHVFGTRNHYGKVDVYAGTFNGILKEAGVDYTETFETPTIMATFKAMLHDWTNEGFDPFAA  146
Burkholderia_multivorans_ATCC_ MATLETLD---LSAGFAADLVQATQADAVTGYRRFQLGEFAFQRDEYFVKISWPAK-GQTRTHAIPADAFLRAMMRDVAWGFFYGWVNFDHVFGTRNHYGKVDVYAGTFNGILKEAGVDYTETFETPTIMATFKAMLHDWTNEGFDPFAA  146
Burkholderia_ambifaria_MEX-5   MATLETLD---LSAGFAADLVQATEADAVTGYRRFQLGAFAFQRDEYFVKISWPAK-GSTRTHAIPADAFLRAMMRDVAWGFFYGWVNFDHVFGTRNHYGKVDVYAGTFNGILKEAGVDYTETFETPTIMATFKAMLHDWTNEGFDPFAA  146
Burkholderia_ambifaria_MC40-6  MATLETLD---LSAGFAADLVQATEADAVTGYRRFQLGAFAFQRDEYFVKISWPAK-GSTRTHAIPADAFLRAMMRDVAWGFFYGWVNFDHVFGTRNHYGKVDVYAGTFNGILKEAGVDYTETFETPTIMATFKAMLHDWTNEGFDPFAA  146
Burkholderia_ambifaria_AMMD    MATLETLD---LSAGFAADLVQATEADAVTGYRRFHLGAFAFQRDEYFVKISWPAK-GSTRTHAIPADAFLRAMMRDVAWGFFYGWVNFDHVFGTRNHYGKVDVYAGTFNGILKEAGVDYTETFETPTIMATFKAMLHDWTNEGFDPFAA  146
Burkholderia_ambifaria_IOP40-1 MATLETLD---LSAGFAADLVQATEADAVTGYRRFHLGAFAFQRDEYFVKISWPAK-GSTRTHAIPADAFLRAMMRDVAWGFFYGWVNFDHVFGTRNHYGKVDVYAGTFNGILKEAGVDYTETFETPTVMATFKAMLHDWTNEGFDPFAA  146
Burkholderia_sp._383           MATLDTLD---TSAGFAADLVRSTEADAVTGYRRFQLGAFEFQRDEYFVKICWPAK-GQTRTHAMPADAFLRAMMRDVAWGFFYGWVNFDHVFGTRNHYGKVDMYAGTFNGILKDAGVDYTETFETPTIMATFKAMLHDWTNESFDPFAA  146
Variovorax_paradoxus_S110      MNAPAELA-----------KVIASQPDATLGYKDFSLGSFGFRRDEYFVHITWKTRDGRPMSHTMDAGSYLRALMRDVAWGFFYGWVNFDNVFGTINHYDSVDLYAGSFNGTMKDAGIDLLENFPTAQIRATFEAMLDDWTNESFDPFAA  139
Photorhabdus_luminescens_subsp ----------------MNTNLKCTIDNTETGYQTFTAGHFTFDRCEYFAYISCPTG-----EHMIPIDDYLKALMRDIAWGFFYGTVAFDDVLGTTNHYGNVDLYMGSFHKEWTRAGRDYKENFKSDELMAIFKAMISDWTNTGFDPFAA  129
Sphingomonas_sp._strain_TTNP3  MAMSEALEIIDFGDSKARTDTEHLAINNETGYRSFRAGGFTFTRDEYFARLTWPGG-----SHIIPIDAFLRAMMRDVAWGFFYGVVNFDHVFGTINHYGEVTMFAGRFNDAYRNAGRDHEERFKSSALMAVFKDILSDWTVEGYDPFAA  145
                               1.......10........20........30........40........50........60........70........80........90.......100.......110.......120.......130.......140.......150
```

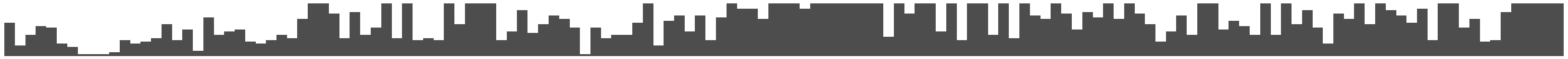

```
                               * *** .:* * *::   * * *  ::*  *: .*  :*       :** * ** * :* :    *:: :: *:.*: :****:::****. **   * :***:**.:**: ***** ***:*::*:* **  * . . * *::  :.**
Pseudomonas_sp._1-7            PEETGSAFGRKNGENLECIERFRIATKRMPGLQDDSPLRNDL---PVNRQFADVSQDEPEVHAAEGFEGELHAFSLFKYLSRSDVTWNPSVTSVCKASLFCPTTEEFILPVFHGNDRVEWFIQMSDEIVWDVADKDDGSPRARITMRAGD  293
Pseudomonas_sp._WBC-3          PEETGSAFGRKNGENLECIERFRIATKRMPGLQDDSPLRNDL---PVNRQFADVSQDEPEVHAAEGFEGELHAFSLFKYLSRSDVTWNPSVTSVCKASLFCPTTEEFILPVFHGNDRVEWFIQMSDEIVWDVGDKDDGNPRARITMRAGD  293
Pseudomonas_putida             PQETGTAFGRKNGENLEAIERFRIATKRMPGLPDDSPLRNDL---EINRHFADVSQDEPQIHAAEGFEGELHAFSLFKYLSRSDVTWNPSVTSVCKASLFCPTTEEFMLPVFHGNDRVEWFLQMSDDIVWDVADKDEGNPRARITMRPGD  293
Pseudomonas_sp._NyZ402         PQETGTAFGRKNGENLEAIERFRIATKRMPGLPDDSPLRNDL---EINRHFADVSQDEPQIHAAEGFEGQLHAFSLFKYLSRSDVTWNPSVTSVCKASLFCPTTEEFMLPVFHGNDRVEWFLQMSDDIVWDVADKDDGNPRARITMRPGD  293
Pseudomonas_fluorescens_ACB    PAETGTAFGRKNGENIEAIERFRVATRRMPGLADDSPLRNDL---PVNRQFADVVQDEPEVHAADGFEGELHAFSLYKYLSRSDVSWNPSVTSVCGASLFCPTTEEFILPIVHGNDRIEWFLQLSDEIIWDIGDKDDGDPRARITMRAGD  295
Pseudomonas_aeruginosa_PA7     PEETGSAFGRKHGDNTEAIERFRIATRRMPGLPDDSPLRDDL---PVNRQFLDVPQDEPELHPAEGYEGELHAFNLFKYLSRSDVTWNPSVSSVCKASLFCPTTEEFILPVFHGNDRVEWFLQLSDEIVWDVADKDDGTPRARVIMRAGD  293
Burkholderia_sp._CCGE1002      PEETGTAFGRKHGDNDAAIERTRIATRRMPGLEGDSPLRDDL---PVNRAFADVTQDDPEVHVEPGFEGQLHAFNLFKYLSRSDVTWNPSVTSVCGQSLFCPTTEEYILPVFHGNDRVEWFLQLSDEIVWDIGDKNTGAPRARITMRAGD  293
Burkholderia_sp._H160          PEETGTAFGRKHGDNDAAIERTRIATRRMPGLEGDSPLRDDL---PVNRAFADVTQDDPEVHVEPGFEGQLHAFNLFKYLSRSDVTWNPSVTSVCGQSLFCPTTEEYILPVFHGNDRVEWFLQLSDEIVWDIGDKNTGAPRARITMRAGD  293
Burkholderia_cenocepacia_HI242 PEETGTAFGRKHGENGAAIERTRIATRRMPGLEGDSPLRDEL---PVNRAFLDVTQDEPEVHVEPGFDGELHAFNLFKYLSRSDVTWNPSVTSVCGRSLFCPTTEEFILPVFHGNDRVEWFLQLSDEIVWDIGDKNTGAPRARITMRAGD  293
Burkholderia_cenocepacia_J2315 PEETGTAFGRKHGDNGAAIERTRIATRRMPGLEGDSPLRDDL---PVNRAFLDVAQDEPEVHVEPGFEGELHAFNLFKYLSRSDVTWNPSVTSVCGRSLFCPTTEEFILPVFHGNDRVEWFLQLSDEIVWDIGDKNTGAPRARITMRAGD  293
Burkholderia_multivorans_CGD2M PQETGSAFGRKHGENDSAIERTRIATRRMPGLEGDSPLRDAL---PVNRAFLDVDQDEPEVHVEPGFEGELHAFNLFKYLSRSDVTWNPSVTSVCGRSLFCPTTEEYILPVFHGNDRVEWFLQLSDEIVWDIGDKNTGAPRARITMRAGD  293
Burkholderia_multivorans_CGD1  PQETGSAFGRKHGENDSAIERTRIATRRMPGLEGDSPLRDAL---PVNRAFLDVDQDEPEVYVEPGFEGELHAFNLFKYLSRSDVTWNPSVTSVCGRSLFCPTTEEYILPVFHGNDRVEWFLQLSDEIVWDIGDKNTGAPRARITMRAGD  293
Burkholderia_multivorans_ATCC_ PEETGSAFGRKHGENDSAIERTRIATRRMPGLEGDSPLREAL---PVNRAFLDVDQDEPEVHVEPGFEGELHAFNLFKYLSRSDVTWNPSVTSVCGRSLFCPTTEEYILPVFHGNDRVEWFLQLSDEIVWDIGDKNTGAPRARITMRAGD  293
Burkholderia_ambifaria_MEX-5   PEETGTAFGRKHGENGAAIDRTRIATRRMPGLEGDSPLREEL---PVNRAFRDVGQDEPEVHVKPGFEGELHAFNLFKYLSRSDVTWNPSVTSVCGRSLFCPTTEEFILPVFHGNDRVEWFLQLSDEIVWDIGDKNTGAPRARITMRAGD  293
Burkholderia_ambifaria_MC40-6  PEETGTAFGRKHGENGAAIERTRIATRRMPGLEGDSPLRDEL---PVNRAFRDVGQDEPEVHVKPGFEGELHAFNLFKYLSRSDVTWNPSVTSVCGRSLFCPTTEEFILPVFHGNDRVEWFLQLSDEIVWDIGDKNTGAPRARITMRAGD  293
Burkholderia_ambifaria_AMMD    PEETGTAFGRKHGENGAAIERTRIATRRMPGLEGDSPLRDEL---PVNRAFRDVGQDEPEVHVKPGFEGELHAFNLFKYLSRSDVTWNPSVTSVCGRSLFCPTTEEFILPVFHGNDRVEWFLQLSDEIVWDIGDKNTGAPRARITMRAGD  293
Burkholderia_ambifaria_IOP40-1 PEETGTAFGRKHGENGAAIERTRIATRRMPGLEGDSPLRDEL---PVNRAFRDVGQDEPEVHVKPGFEGELHAFNLFKYLSRSDVTWNPSVTSVCGRSLFCPTTEEFILPVFHGNDRVEWFLQLSDEIVWDIGDKNTGAPRARITMRAGD  293
Burkholderia_sp._383           PEETGTAFGRKHGDNGSAIERTRIATRRMPGLEGDSPLRDDL---PVNRAFVDVAQDEPEVHVEPGFEGELHAFNLFKYLSRSDVTWNPSVTSVCGRSLFCPTTEEFILPVFHGNDRVEWFLQLSDEIVWDIGDKNTGAPRARVTMRAGD  293
Variovorax_paradoxus_S110      PQETGSPYGRKSGNNTAKITRARELAKRCVGLKGDLDLRSDARGAPVNRAFADVPQAQPELHPEPGFENEVHAFNLFGFLSRSQVTWNPSFTSVVKHSYMCPTTEEHILPIIHGNDRVEWFFQMTDEIHWDCGDKNTGRPMARVIMKAGD  289
Photorhabdus_luminescens_subsp PQETGVAWGRKNGNEDRLIGRKREVAKRMVGIPGDAPLRSDDNGHPVNRAFMDVNQEQPVVKAEPGFEGELHAFNLFGYLSRSDMTWNPSVVSVVRQSLFCPTTEEFILPIDHGNDRAEWFIQLSDEIAWDIKDGKSSSPLAKVTMRAGD  279
Sphingomonas_sp._strain_TTNP3  PMETGLPWGIKNGNNDEAISRQRVTARRMVGLPGDTPVRTDANGFPVNRQFADVPQEQPVVEAEPGFEAEVSAYNLFGYLSRSDVTWNPSVCSVVGDSLFCPTSEEFILPVEHGNDRCEWFLQLSDEIVWDVKDKESGKPRARVTARAGD  295
                               .......160.......170.......180.......190.......200.......210.......220.......230.......240.......250.......260.......270.......280.......290.......300
```

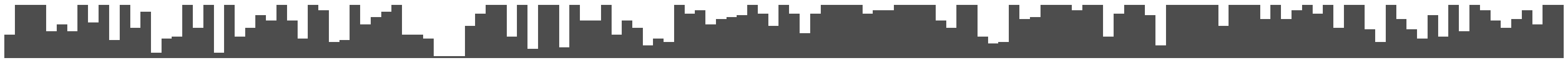

CLUSTAL 2.0.11 MULTIPLE SEQUENCE ALIGNMENT

File: F:/hqdB sequence alignment.ps **Date: Fri Apr 01 15:19:56 2011**


```
                                 :..**.  **** *.*****:****.:*.:      .*     *: *
Pseudomonas_sp._1-7              VCAMPADIRHQGYSTKRSMLMVWENATPNLPHLYESGELKPYPIEF   339
Pseudomonas_sp._WBC-3            VCAMPADIRHQGYSTKRSMLMVWENATPNLPHLYESGELKPYPIEF   339
Pseudomonas_putida               ICAMPADIRHQGYSTKRSMLMVWENATPNLPERYESGELTPYPIEF   339
Pseudomonas_sp._NyZ402           ICAMPADIRHQGYSTKRSMLMVWENATPNLPERYESGELTPYPIEF   339
Pseudomonas_fluorescens_ACB      ICAMPADIRHQGYSTKRSMLLVWENATPNLPQRYESGELKPYPVEF   341
Pseudomonas_aeruginosa_PA7       IAAMPADIRHQGYSTKRSMLLVWENATPNLPQRYESGELKPYPVDF   339
Burkholderia_sp._CCGE1002        ICAMPADIRHQGYSTKRSMLLVWENATPNLPKRYESGELKPYPVDF   339
Burkholderia_sp._H160            ICAMPADIRHQGYSTKRSMLLVWENATPNLPKRYESGELKPYPVDF   339
Burkholderia_cenocepacia_HI242   ICAMPADIRHQGYSTKRSMLLVWENATPDLPQRYESGELAPYPIAF   339
Burkholderia_cenocepacia_J2315   ICAMPADIRHQGYSTKRSMLLVWENATPDLPQRYESGELAPYPIAF   339
Burkholderia_multivorans_CGD2M   ICAMPADIRHQGYSTKRSMLLVWENATPDLPQRYESGELAPYPIEF   339
Burkholderia_multivorans_CGD1    ICAMPADIRHQGYSTKRSMLLVWENATPDLPQRYESGELPPYPIEF   339
Burkholderia_multivorans_ATCC_   ICAMPADIRHQGYSTKRSMLLVWENATPDLPQRYESGELAPYPIEF   339
Burkholderia_ambifaria_MEX-5     ICAMPADIRHQGYSTKRSMLLVWENATPDLPQRYESGELAPYPIEF   339
Burkholderia_ambifaria_MC40-6    ICAMPADIRHQGYSTKRSMLLVWENATPDLPQRYESGELAPYPIEF   339
Burkholderia_ambifaria_AMMD      ICAMPADIRHQGYSTKRSMLLVWENATPDLPQRYESGELAPYPIAF   339
Burkholderia_ambifaria_IOP40-1   ICAMPADIRHQGYSTKRSMLLVWENATPDLPQRYESGELAPYPIAF   339
Burkholderia_sp._383             ICAMPADIRHQGYSTKRSMLLVWENATPNLPQRYESGELAPYPIAF   339
Variovorax_paradoxus_S110        MAAMPAYCRHQGFSPKRSMLLVWENGSPSLVYEIQKGESPEIPVEF   335
Photorhabdus_luminescens_subsp   IAAMPGDIRHQGMSPKRSMLLVWENGSPEIMDMIKDGTIPMVPVTF   325
Sphingomonas_sp._strain_TTNP3    ICCMPADIRHQGYSTKRSMLLVWENGSPKIPQMIADGTAPVVPVTF   341
                                 .......310.......320.......330.......340......
```

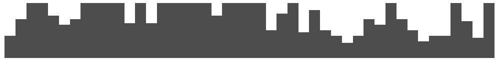

Supplement: Additional file 3 — Multiple sequence alignment performed by ClustalW 2 of the sequence of HqdB with sequences retrieved by BLAST search. Shown is the original multiple sequence alignment from which Figure 4B has been rendered. [file 2191-0855-1-8-S3.PDF]
